# Polarization Property Associated with Surface Plasmon Resonance in a Palladium Thin-Film Coated Aluminum Grating in a Conical Mounting and Its Application to Hydrogen Gas Detection

**DOI:** 10.3390/s24061990

**Published:** 2024-03-20

**Authors:** Toyonori Matsuda, Isao Tsunoda, Shinichiro Koba, Yu Oshiro, Hiroyuki Odagawa

**Affiliations:** 1 Institute of National College of Technology, Kumamoto College, Kumamoto Campus, 2659-2 Suya, Koshi 861-1102, Kumamoto, Japan; 2 Institute of National College of Technology, Kumamoto College, Yatsushiro Campus, 2627 Hirayamashin-Machi, Yatsushiro 866-8501, Kumamoto, Japan

**Keywords:** hydrogen gas detection, palladium thin-film, surface plasmon resonance (SPR), aluminum diffraction grating, conical mounting, Stokes parameters

## Abstract

We have investigated a polarization property of the (specularly) reflected light from an aluminum grating, coated with a palladium (Pd) thin-film on its surface. The polarization property, which is associated with surface plasmon resonance (SPR), and occurs in the Pd thin-film on the aluminum grating in a conical mounting, is observed as a rapid change in the normalized Stokes parameter $s_{3},$ around the resonance angle, $\theta_{sp}$, at which point, SPR occurs. The sensing technique used the rapid change in $s_{3}$ to allow us to successfully detect a small change in the complex refractive index of the Pd thin-film layer upon exposure to hydrogen gas, with a concentration near the lower explosion level. Experimental results showed that the sensing technique provided a sensitive and stable response when the Pd thin-film layer was exposed to gas mixtures containing hydrogen at concentrations of 1 to 4% (by volume) in nitrogen.

## 1. Introduction

Hydrogen has attracted much attention as a clean, sustainable, and abundant energy source. However, hydrogen is a flammable gas and becomes explosive when its concentration exceeds 4% (in terms of volume) in air (the lower explosive limit, LEL). Therefore, the use of hydrogen, including its production, storage, and transportation, involves the risk of explosion. For this reason, sensors for monitoring hydrogen concentration, or detecting hydrogen leaks, are indispensable, and various types of hydrogen sensors have been actively developed [1].

Among hydrogen sensors, optical approaches including fiber optics have promising advantages, such as the ability to operate in explosive environments due to electrical isolation and immunity from electromagnetic interference [2,3]. Surface plasmon resonance (SPR) sensors, which are a type of optical hydrogen sensor, have been studied for a significant period of time [4]. SPR sensors are associated with the excitation of propagating surface plasmons, along a metal–dielectric interface, using an optical beam [5]. As the occurrence conditions of SPR strongly depend on the refractive index of the dielectric and the complex refractive index of the metal, SPR has been used for refractive index sensing in various fields, including gas detection [6,7]. There have been a large number of reports concerning the application of SPR sensors to hydrogen gas detection. The SPR sensors are mainly classified into three types, based on couplers, to excite propagating surface plasmons [7], prism couplers, optical waveguide couplers, and grating couplers. An SPR sensor with a glass prism (Kretschmann–Raether configuration), upon which, a palladium (Pd) layer is deposited, was for the first time proposed for the detection of hydrogen gas by Chadwick and Gal [8]. Then, a prism coupler type SPR sensor (Otto configuration) was reported, within which, a glass prism was constructed with an intermediate layer of silica and a sensing layer of Pd on top [9]. As SPR hydrogen sensors with a waveguide coupler, a channel waveguide [10], and an optical fiber [11] were proposed, within which, a thin Pd film was coated on a portion of the core, from which, the cladding was then removed. Furthermore, various types of optical fiber hydrogen sensors [3], such as multimode fibers [11,12], fiber gratings [13], and tapered fibers [14], have been actively developed, owing to their potential for remote and multiplex sensing [15]. A metal diffraction grating, coated with a thin Pd film on its surface, has been proposed as a hydrogen SPR sensor [16]. SPR hydrogen sensors are based on a technology that integrates refractive index sensing with hydrogen sensitive materials such as Pd [3]. Hydrogen sensitive materials that sensitively, selectively, and quickly convert the absorption or adsorption of hydrogen into a change in their own refractive index have been extensively studied as transducers in the following SPR hydrogen sensors [3,17]: Pd alloys [18,19], multilayers including a Pd layer [12,20], Pd composite films [21], Pd nanofilms on photonic crystal [22], etc. Furthermore, an accurate and simple technique for detecting the change in optical properties, caused by the exposure of a hydrogen sensitive material to hydrogen gas, could be essential for the practical application of SPR hydrogen sensors. This requires the development of a measurement technique to accurately detect minute changes in the (complex) refractive index of a hydrogen sensitive material with a straightforward optical configuration, based on SPR sensing [16].

Here, we discuss an efficient technique that allowed us to detect small changes in the complex refractive index of a Pd thin-film upon exposure to hydrogen gas by using SPR in a metal gating. Given that we examined a hydrogen gas detector, we considered an aluminum diffraction grating, coated with a Pd thin-film on its surface. The Pd thin-film layer serves two purposes [10]: it provides a coupler to excite surface plasmons and a transducer to convert hydrogen exposure into a change in its own complex refractive index. Thus, SPR occurring in the Pd thin-film coated aluminum grating includes the information of a small change in the complex refractive index of the Pd thin-film layer due to hydrogen gas exposure. To accurately and efficiently detect the complex refractive index change in the Pd thin film layer, we applied an SPR sensing technique that uses a polarization property of (specularly) light reflected from a metal grating [23]. Regarding this sensing technique, a metal grating is arranged in a conical mounting, where the plane of incidence is not perpendicular to its grooves [24,25]; then, the normalized Stokes parameter, $s_{3}$, of the reflected light (which means that the intensity difference between the right- and left-circularly polarized components) is measured. When a metal grating in a conical mounting is illuminated with TM (*p*)-polarized light whose electric field is parallel to the plane of incidence, $s_{3}$ rapidly changes with the angle of incidence around the resonance angle $\theta_{sp}$, at which point, SPR occurs. The rapid change in $s_{3}$ results in the following interesting features of refractive index sensing: $\theta_{sp}$ is determined as the zero-crossing point on the $s_{3}$ curve (incident angle dependence of $s_{3}$), and a small change in the refractive index of a sample is detected by measuring $s_{3}$ under the fixed angle of incidence, which is $\theta_{sp}$. The effectiveness of the SPR sensing technique, using the rapid change in $s_{3},$ has been demonstrated by experiments comprising the following: detection of the refractive index difference among gaseous samples, including H_2_, O_2_, N_2_, and CO_2_ [23], and ethanol concentration measurements of ethanol–water solutions [26]. In these experiments, the variations in a real refractive index were detected using an aluminum grating in a conical mounting.

In this study, we deposited an approximately 50 nm thick Pd thin-film on the surface of a commercially available aluminum grating, with a groove density of 2400 lines/mm. Then, we investigated the polarization property of the reflected light from the Pd thin-film coated aluminum grating in a conical mounting when TM-polarized light, with a wavelength of 672 nm, illuminated it. As a result, we revealed a rapid change in $s_{3},$ with a steep slope of around $\theta_{sp}$, which is associated with the sharp SPR occurring in the Pd thin-film layer. Notably, the steep slope where the rapid change in $s_{3}$ occurs makes a small change in the complex refractive index of the Pd thin-film layer significantly vary $s_{3}$ in the vicinity of $\theta_{sp}$. Therefore, measuring the variation in $s_{3}$ allowed us to detect hydrogen gas near the LEL. We experimentally showed that the SPR sensing technique, measuring the variation in $s_{3}$, sensitively and stably, detected a gas mixture of hydrogen with a 4% (by volume) concentration in nitrogen, and provided a good response to the change in hydrogen concentration, with 1 to 4% of nitrogen.

## 2. Preparation for Experiments

### 2.1. Pd Thin-Film Coated Aluminum Grating

**Figure A1:**
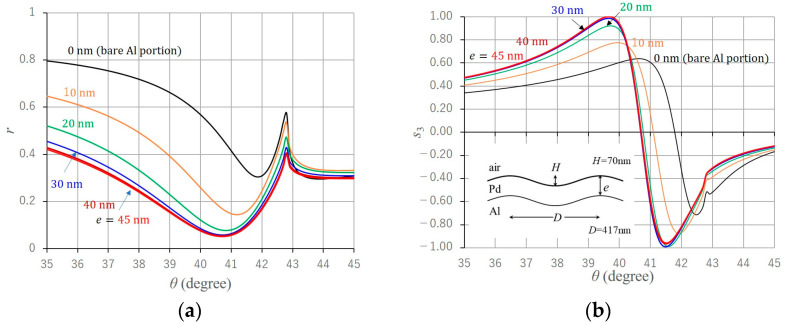
Numerical results concerning the effect of the Pd thin-film thickness on SPR properties. (**a**) Reflectance r and (**b**) Stokes parameter $s_{3}$ of the reflected light on the Pd thin-film coated aluminum grating with different thicknesses, $e=0\;\left(bareAlgrating\right),\;10,\;20,\;30,\;40,or\;45\;nm$.

We examined a metal diffraction grating coated with a Pd thin-film which served as a coupler to excite surface plasmons and a transducer to convert hydrogen exposure to a change in its complex refractive index. We have observed that UV holographic aluminum gratings from Edmund Optics, Inc., Tokyo, Japan which have a shallow groove depth of several tens of nanometers, exhibit sharp SPR characteristics [23,26]. In this study, we used a UV holographic aluminum grating, with a groove density of 2400 lines/mm (Edmund Optics, Inc., stock no. 43776), as a metal diffraction grating, and we coated its surface with a Pd thin-film. We carried out a computer simulation to investigate the polarization property of the reflected light from the holographic aluminum grating coated with a Pd thin-film layer (Figure A1 in Appendix A). As a result, we estimated that the Pd thin-film thickness should be approximately 45 nm as it provides a sharp SPR that can be used for hydrogen detection.

**Figure 1:**
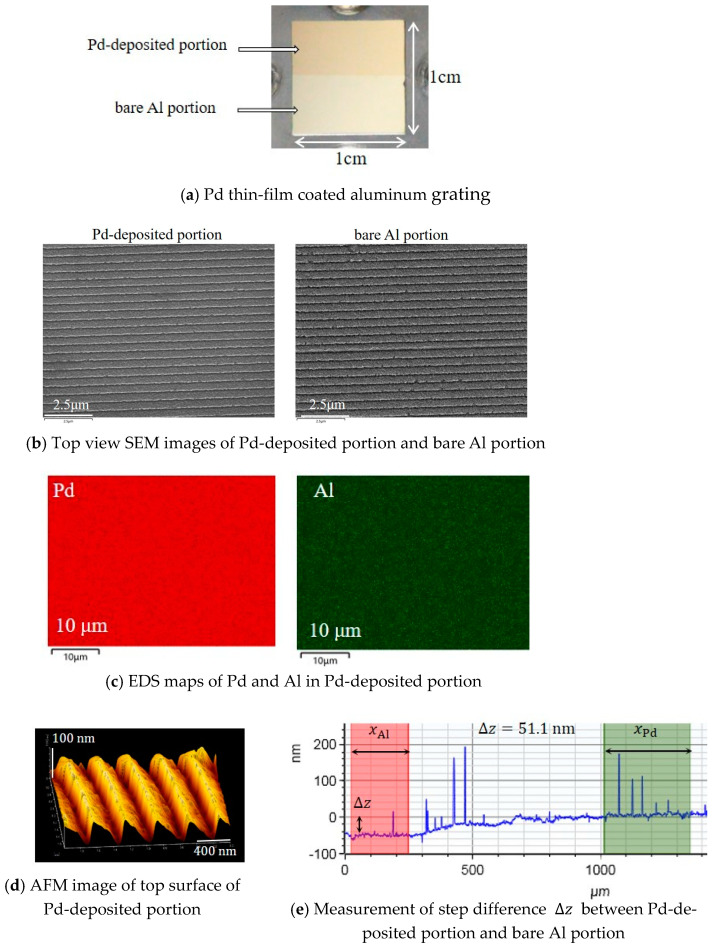
Deposition of Pd thin-film on the upper half of the surface of the aluminum grating. (**a**) Appearance of Pd thin-film coated aluminum grating; (**b**) top-view SEM images of Pd-deposited portion and bare Al portion; (**c**) EDS maps of Pd and Al in the Pd-deposited portion; (**d**) AFM image of the Pd-deposited portion; and (**e**) step difference measurement between the Pd-deposited portion and bare Al portion.

We deposited a Pd thin-film on a half portion of the surface of the holographic aluminum grating with an Nd-YAG pulsed laser deposition (PLD) system (Pascal Co., Ltd., Osaka, Japan, PLD-system). The conditions of the PLD were as follows: lamp power of 29 J and a pulsed laser energy density of 80 mJ/cm^2^; wavelength of 266nm; pulse width of less than 2 ns; pulse repetition rate of 10 Hz; deposition rate of 15 Å/min; deposition time of 30 min. As shown in Figure 1a, the upper portion of the grating surface coated with the Pd thin-film is referred to as the “Pd-deposited portion”. The lower portion, where a Pd thin film was not deposited, owing to masking in the deposition process, is referred to as the “bare Al portion”. We observed the surface of the holographic aluminum grating was coated with the Pd thin-film. Figure 1b shows the SEM images of the Pd- deposited portion and the bare Al portion, which were measured using a scanning electron microscope (JEOL, Tokyo, Japan, JSM-7001F). The SEM images indicate that the periodic structures in the Pd-deposited portion are almost the same as those in the bare Al portion. We then examined the distribution of Pd and Al (aluminum) elements in the Pd-deposited portion with Energy Dispersive X-ray Spectroscopy (EDS) analysis. In the EDS maps shown in Figure 1c, Pd is observed to be uniformly distributed in the Pd-deposited portion, whereas Al is attributed to the aluminum grating under the Pd thin-film. We examined the top surface of the Pd-deposited portion with an atomic force microscope (AFM) (SII Nano Technology Inc., Chiba, Japan, NanoNavi E-sweep). As shown in Figure 1d, the AFM image illustrates the periodic structures, with periods of around 400 nm and a corrugation depth of about 70 nm. Finally, we measured the step difference amount between the Pd-deposited portion and the bare Al portion with a surface stylus profiler (Bruker, Yokohama, Japan, Dektak XT). In Figure 1e, the difference between the average height measured in the Pd-deposited region ($x_{Pd}$) and that of the bare Al region ($x_{Al}$) was ∆z=51.1 nm, and thus, we estimated the (average) thickness of the Pd thin-film layer to be e= 50 nm. Prominent signals in the measured data were considered to be due to cracks on the top surface of the Pd thin-film. In light of the above, the periodic structures of the Pd thin-film were thus fabricated on the holographic aluminum grating.

**Figure 2:**
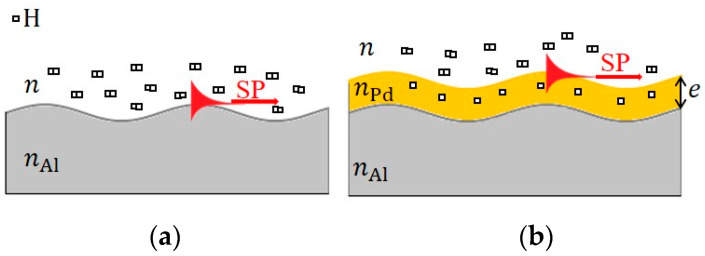
Cross sections of (**a**) the bare Al portion, (**b**) the Pd-deposited portion on the aluminum grating, and the surface plasmons (SP) excited in each portion.

Here, we note that SPR occurs in the bare Al portion and in the Pd-deposited portion of the aluminum grating. Figure 2 illustrates the cross-sections of the bare Al portion and the Pd-deposited portion which were exposed to hydrogen gas in air. Surface plasmons in the bare Al portion are excited along the surface of the aluminum grating, and the resultant SPR depends on the refractive index of the upper region of the bare Al portion, n, and the complex refractive index of Al, $n_{Al}$. When the bare Al portion is exposed to hydrogen gas, a significant change in the behavior of the SPR does not appear because the refractive index of hydrogen gas is close to that of air and the complex refractive index of Al is largely unaffected due to hydrogen gas exposure. On the other hand, SPR in the Pd-deposited portion is caused by the excitation of surface plasmons, supported by the Pd thin-film layer, if its thickness e is thick (for instance, 50 nm). When exposed to hydrogen gas, the Pd thin-film layer selectively absorbs hydrogen as a hydrogen sensitivity material, resulting in a change in the complex refractive index of the Pd thin-film layer $n_{Pd}$ [11,17]. Therefore, SPR in the Pd-deposited portion is significantly affected by hydrogen gas exposure. 

### 2.2. Optical Configuration and Experimental Setup

**Figure 3:**
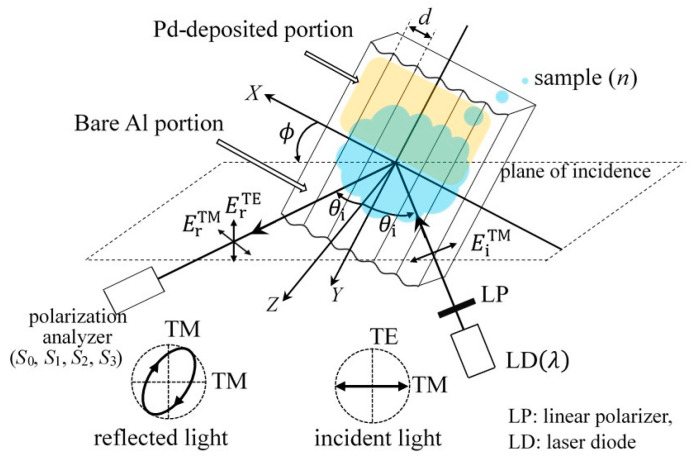
Pd thin-film coated aluminum grating in a conical mounting and optical configuration for hydrogen gas detection.

Figure 3 illustrates the optical configuration to investigate the polarization property of the reflected light from the bare Al portion or the Pd-deposited portion described in Section 2.1. The aluminum grating is periodic, with a period of d=417 nm in the X direction, its grooves are parallel to the Y direction, and the grating normal lies in the Z direction. The aluminum grating is arranged in a conical mounting where the angle between the plane of incidence and the X axis is denoted by the azimuthal angle, ϕ. The front area of the grating surface is filled with the gas sample, with a refractive index of n. As a light source, we used a laser diode (LD) module (Edmund Optics Inc., Stock #38-922) with a continuous-wave beam, with a wavelength of λ=672 nm, and power of 3 mW. The output light from the LD module becomes TM polarized after passing through a linear polarizer (LP), the transmission axis of which is parallel to the plane of incidence. The TM-polarized light illuminates d the surface of the aluminum grating at the angle of incidence $\theta_{i}$, which is measured from the Z axis. By moving the light-source part and light-receiving part up and down simultaneously, the incident light illuminates either the bare Al portion or the Pd-deposited portion, and the reflected light is then received by a polarization analyzer.

SPR in a metal grating, in a conical mounting, has been studied [27,28,29,30,31,32], and it has interesting features for refractive index sensing [25,28,31,32]. In particular, the polarization property of the reflected light associated with SPR is attractive [23]. When the TM-polarized light is incidental on either the Pd-deposited portion or the bare Al portion on the aluminum grating in the conical mounting, TM and TE components appear in the diffracted light. Here, TM and TE mean that the relevant magnetic and electric fields are transverse to the Z axis, respectively. The reflected light from the Pd-deposited portion or the bare Al portion includes the TM- and TE-components; then, it becomes elliptically polarized in general for the TM-polarized incidence. To examine the polarization state of the reflected light, we measured its Stokes parameters $S_{0}$ to $S_{3}$ with a polarimeter (Thorlabs Inc., Tokyo, Japan, PAX1000VIS). As a quantity for detecting hydrogen gas, we employed the Stokes parameter that was normalized by the intensity of the reflected light $I\left(=S_{0}\right)$:

(1) $$s_{3}=\frac{S_{3}}{I}=2\frac{E_{r}^{TE}/E_{r}^{TM}}{1+{\left(E_{r}^{TE}/E_{r}^{TM}\right)}^{2}}\sin\delta .$$

Here, $E_{r}^{TM}$ and $E_{r}^{TE}$ are the amplitudes of the TM and TE components of the electric field of the reflected light, respectively, and $\delta =\delta_{TE}-\delta_{TM}$ is the phase difference between them. The normalized Stokes parameter $s_{3}$, which indicates the difference in intensity between the right- and left-circularly polarized components, varies from 1 (right-circular polarization) to −1 (left-circular polarization) via 0 (linear polarization). We also evaluated the phase difference δ and the amplitude ratio $E_{r}^{TE}/E_{r}^{TM}$ from the measured Stokes parameters $S_{0}$ to $S_{3}$ to examine the polarization property associated with SPR from the behavior of the TM and TE components of the reflected light.

In this study, we examined SPR occurring in the Pd-deposited portion, or in the bare Al portion, of the aluminum grating when the reflected light (or the zeroth-order diffracted mode) only propagates, and when the TM component of the −1st-order evanescent mode in the diffracted light couples with surface plasmons. In the optical configuration shown in Figure 3, the zeroth-order diffracted mode is propagated, and all other diffracted modes are evanescent when the following relation is satisfied for m=−1 [31]:

(2) $${\hat{\alpha }}_{m}^{2}+{\hat{\beta }}^{2}>n^{2},$$

where ${\hat{\alpha }}_{m}=n\sin\theta_{i}\cos\phi +m\frac{\lambda }{d}$ and $\hat{\beta }=n\sin\theta_{i}\sin\phi$ are the propagation constants in the X and Y directions of the mth-order evanescent mode that are normalized by the wave number of the incidental light, respectively. We chose the grating period, d=417 nm, and the wavelength of incidental light, λ=672 nm, in the experimental setup so that Equation (2) was satisfied.

SPR in a metal grating occurs when a phase matching condition for the coupling of the TM component, of an evanescent mode, in diffracted light with surface plasmons, is satisfied; that is, the wave vector of the evanescent mode coincides with that of the surface plasmon wave [33]. The phase matching condition for the coupling of the −1st-order evanescent mode with the surface plasmon wave is expressed as follows [31]:

(3) $${\left(Re\left[{\hat{k}}_{sp}\right]\right)}^{2}={\hat{\alpha }}_{-1}^{2}+{\hat{\beta }}^{2}.$$

Here, ${\hat{k}}_{sp}$ is the propagation constant of the surface plasmon wave normalized by the wave number of the incidental light, and $Re\left[\right]$ denotes a real part of the complex number. Equation (3) indicates that the occurrence of SPR is determined by ${\hat{k}}_{sp}$, the angle of incidence, θ, and the azimuthal angle, ϕ, as the wavelength of the incidental light $\lambda \left(=672\;nm\right)$ and the grating period $d\left(=417\;nm\right)$ are kept constant, as shown in the optical configuration in Figure 3. When the thickness of the Pd thin-film is thick (for instance, 50 nm), ${\hat{k}}_{sp}$ in the Pd-deposited portion is approximated using the following equation [5]:

(4) $${\hat{k}}_{sp}=\frac{nn_{Pd}}{\sqrt{n^{2}+n_{Pd}^{2}}}.$$

Equations (3) and (4) estimate the effect of $n_{Pd}$ on the behavior of SPR in the Pd thin-film coated aluminum grating in a conical mounting.

**Figure 4:**
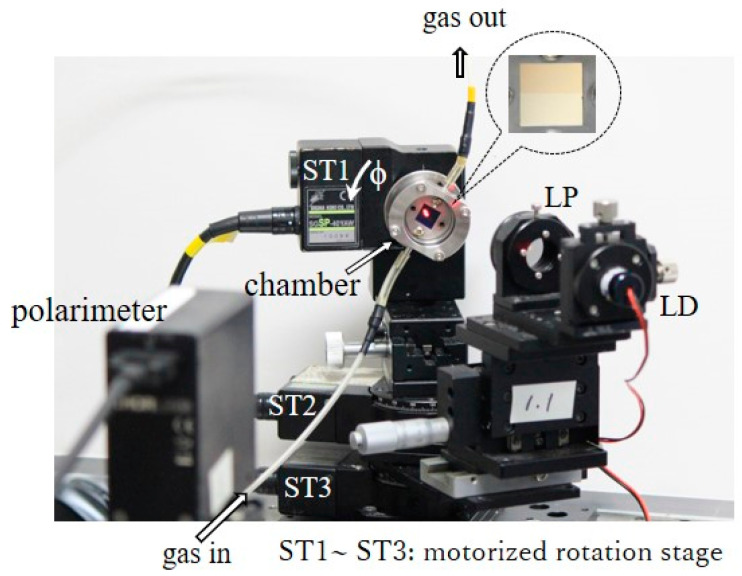
Experimental setup for hydrogen gas detection. Pd thin-film coated aluminum grating is embedded into the chamber; ϕ is varied with a motorized rotation stage (ST1); the angle of incidence is varied using two motorized rotation stages (ST2 and ST3), with a chamber mounted on the axis of rotation.

Figure 4 shows an experimental setup which investigated the polarization property of the reflected light from the bare Al portion or the Pd-deposited portion on the aluminum grating. The aluminum grating was arranged in the chamber so that it could be rotated about its central axis to set the azimuthal angle ϕ with a motorized rotation stage (ST1). The incidental light illuminated either the bare Al portion or the Pd-deposited portion through a glass window of the chamber, and the reflected light was then received by the polarimeter after passing through the glass window. We varied the angle of incidence on the grating surface $\theta_{i}$ (illustrated in Figure 1) with the theta-2 theta scan. The chamber and the incidental light, respectively, were rotated by θ and 2θ, and two of the motorized rotation stages (ST2 and ST3) had the same rotation axis. The relationship between $\theta_{i}$ and θ is given by Snell’s law as $n_{air}\sin\theta =n\sin\theta_{i},$ with $n_{air}$ and n as refractive indices of an air and gas sample, respectively. We therefore refer to θ as the angle of incidence hereinafter. A gas sample was injected into the chamber through a tube from a gas cylinder, and the gas on the chamber was dissipated into the atmosphere.

## 3. Experimental Results and Discussion

We report the experimental results obtained with the experimental setup stated above. The experimental results show that the polarization property of the reflected light associated with SPR in the Pd-deposited portion of the aluminum grating could be available when detecting hydrogen gas of a concentration near the LEL. The experiment was performed in a laboratory, at room temperature, and under atmospheric pressure conditions.

### 3.1. Polarization Property of SPR in Pd-Deposited Portion

**Figure 5:**
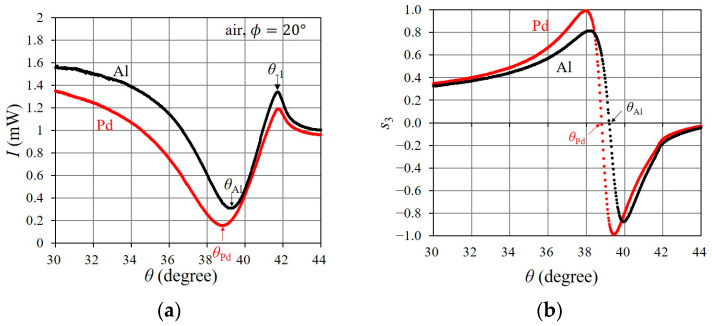
SPR in the bare Al portion and in the Pd-deposited portion on the aluminum grating when ϕ=20,°and when the gas sample is air. (**a**) Intensity of reflected light, I, and (**b**) normalized Stokes parameter, $s_{3}$. “Al” and “Pd” indicate the portion that is illuminated by the incidental light (i.e., bare Al portion and Pd-deposited portion).

We investigated the polarization property of the reflected light associated with SPR occurring in the bare Al portion and in the Pd-deposited portion when a gas sample comprised air. Figure 5a,b show the I and $s_{3}$ of the reflected light from the bare Al portion and the Pd-deposited portion of the aluminum grating, with the azimuthal angle set to ϕ=20°, when θ varied between 30° and 44°. The cut-off for the −1st-order diffracted mode is denoted by $\theta_{-1}=41.72{}^{\circ}$ in Figure 5, and the zeroth-order diffracted mode only propagates and the other diffracted modes are evanescent in the range of θ to less than $\theta_{-1}$. The I and $s_{3}$ curves of the reflected light from the bare Al portion show the occurrence of SPR at $\theta_{Al}=39.19{}^{\circ},$ which is the zero-crossing point of $s_{3}$. The I curve shows the partial absorption of the incidental light as a dip in the vicinity of $\theta_{Al,}$ and $s_{3}$ rapidly fluctuates between a positive maximum value and a negative minimum value via zero at $\theta_{Al}$. The rapid change in $s_{3},$ as well as the absorption dip of I, is caused by the occurrence of SPR [23]. The SPR of $\theta_{Al}=39.10{}^{\circ}$ in the bare Al portion is associated with the coupling of the TM component in the −1st-order evanescent mode with the surface plasmon wave propagating along the surface of the aluminum grating [33]. Next, we describe SPR occurring in the Pd-deposited portion. The I and $s_{3}$ curves of the reflected light from the Pd-deposited portion show the occurrence of SPR at $\theta_{Pd}=38.80{}^{\circ}$ as the rapid change in $s_{3}$ and the absorption dip of I occur. Note that $s_{3}$ fluctuates more rapidly from 1 to −1 via 0 at $\theta_{Pd},$ and the rapid change in $s_{3}$ has a steeper slope around $\theta_{Pd}$. SPR in the Pd-deposited portion is caused by the coupling of the TM component of the −1st-order diffracted evanescent mode with the surface plasmon wave, which is supported by the Pd thin-film layer. Therefore, the occurrence of SPR in the Pd-deposited portion depends on the complex refractive index of the Pd thin-film layer, as expected from Equation (4).

The rapid change in $s_{3,}$ with the steep slope observed in the Pd-deposited portion, has useful features for detecting a small change in the complex refractive index of the Pd thin-film layer. First, $\theta_{sp}$ is determined as the zero-crossing point on the $s_{3}$ curve. The zero-crossing point detection of $\theta_{sp}$ can be accurately and easily implemented, regardless of the sharpness of SPR, such as when the absorption dip in a reflectance curve is small or broad and shallow. Next, the steep slope of the rapid change in $s_{3},$ in the vicinity of $\theta_{sp},$ causes a large variation in $s_{3,}$ in response to a small change in the complex refractive index of the Pd thin-film layer. Therefore, a small change in the refractive index of a sensing sample can be detected by measuring $s_{3}$ under a fixed angle of incidence at $\theta_{sp}$. The measurement of $s_{3}$, which is the intensity difference between the right- and left-circularly polarized components, may be implemented with a simple measuring device, as the reflected light is a monochromatic light with a high degree of polarization.

**Figure 6:**
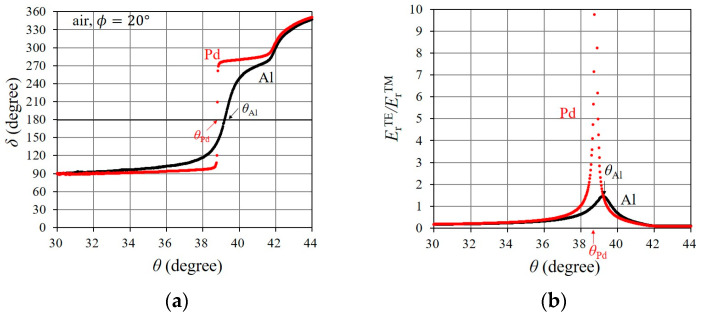
Resonance characteristics of (**a**) δ and (**b**) $E_{r}^{TE}/E_{r}^{TM}$, of reflected light, from the Pd-deposited portion, and the bare Al portion, when SPR occurs. Parameters are the same as in Figure 5.

Here, we describe the occurrence process of the rapid change in $s_{3}$ through the behavior of the TE- and TM-components of the reflected light when SPR occurs in the Pd-deposited portion. This will facilitate a clear understanding of the effectiveness of the SPR sensing technique using the rapid change in $s_{3}$ with the steep slope. Figure 6 shows the δ and $E_{r}^{TE}/E_{r}^{TM}$ curves which correspond to SPR in Figure 5b. With SPR, regarding the bare Al portion, δ varies from 90° to 270° via 180° at $\theta_{Al};$ at the same time, $E_{r}^{TE}/E_{r}^{TM}$ increases in the vicinity of $\theta_{Al}$. Both the phase shift of δ [34] and the increase in $E_{r}^{TE}/E_{r}^{TM}$ [25] result in a rapid change in $s_{3}$ at around $\theta_{Al}$ [23]. With SPR in the Pd-deposited portion, δ very rapidly fluctuates from 90° to 270° at $\theta_{Pd}$. In addition, $E_{r}^{TE}/E_{r}^{TM}$ sharply increases in the vicinity of $\theta_{Pd}$ due to the elimination of $E_{r}^{TM}$, which is caused by the almost total absorption of the TM component of the incidental light by SPR. Thus, the steep slope of the rapid change in $s_{3},$ in the Pd-deposited portion, is caused by the rapid phase shift of δ and a sharp increase in $E_{r}^{TE}/E_{r}^{TM}$; these are largely affected by a change in the occurrence conditions of SPR in the Pd-deposited portion layer.

**Figure 7:**
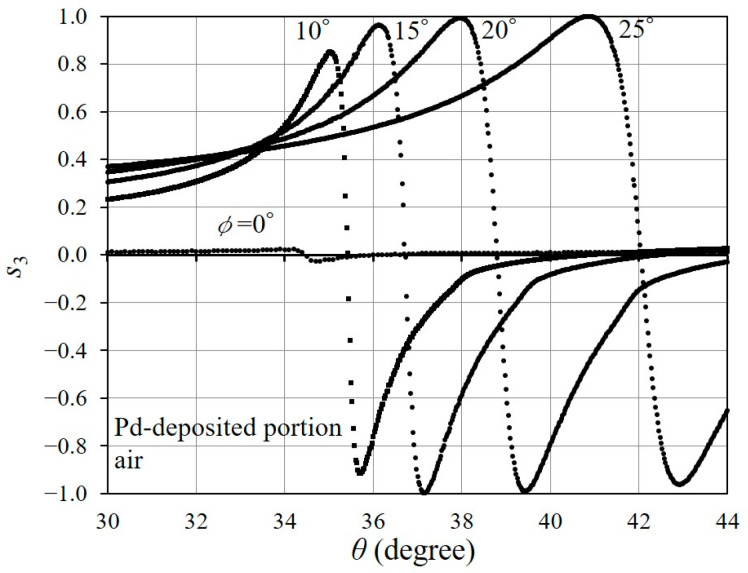
Resonance curves in the Pd-deposited portion for several ϕ values.

The azimuthal angle in a conical mounting ϕ has an effect on the behavior of the rapid change in $s_{3}$. Figure 7 shows the $s_{3}$ curves of the reflected light from the Pd-deposited portion for ϕ=0°,10°,15°, and 25°, in addition to that for ϕ=20°, as shown in Figure 5b. We set the azimuthal angle ϕ as 0°, at which point, the $s_{3}$ curve becomes zero, except for a slight variation in the vicinity of the resonance angle. Then, we chose the ϕ that gave the steeper slope for the rapid change in $s_{3},$ and which, at the same time, caused $s_{3}$ to vary over a wider range. The slope of the $s_{3}$ curve in the vicinity of the resonance angle is related to the sharpness of SPR [23], which affects the sensitivity of the refractive index measurement. In Figure 7, the rapid change in $s_{3}$ for ϕ=20° produces a steep slope around the resonance angle, and the $s_{3}$ fluctuates across a whole range from +1 to −1. Therefore, we used ϕ=20° as the azimuthal angle in the following experiments.

### 3.2. Hydrogen Gas Detection Using Rapid Change in $s_{3}$

We applied a rapid change in $s_{3}$ with the steep slope, which was observed in the Pd deposited portion of the aluminum grating in the conical mounting, at ϕ=20°, to the detection of hydrogen gas. Sample gases comprised mixtures of hydrogen and nitrogen, and they are denoted by $H_{2}\left(C\right);$ the concentrations of hydrogen in nitrogen were C = 1, 2, 3, or 4% (in accordance with volume). Each sample gas $H_{2}\left(C\right)$ was injected into the chamber with a flow rate of 2 L/min, through a tube, from a gas cylinder regulator. The volume of the chamber was approximately 2 mL. The experiment was performed at a temperature of 15.6 °C, the humidity was 40%, and the atmospheric pressure was 1009.3 hPa.

#### 3.2.1. Effect of Hydrogen Gas Exposure on Rapid Change in $s_{3}$

**Figure 8:**
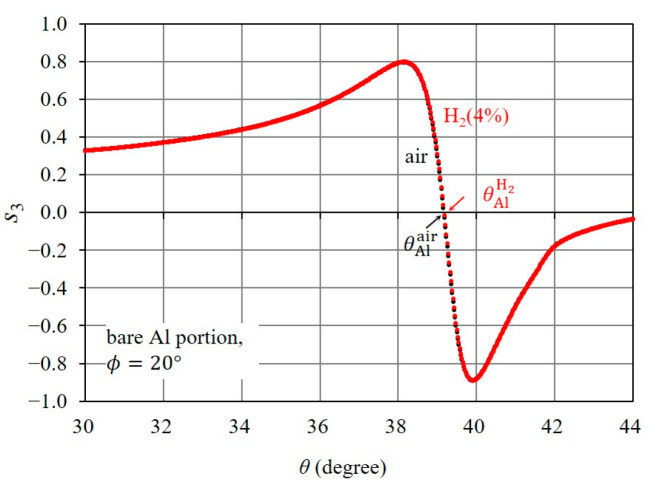
Effect of $H_{2}\left(4\%\right)$ exposure on rapid changes in $s_{3}$ in the bare Al portion. $s_{3}$ curves for $H_{2}\left(4\%\right)$ and air.

**Figure 9:**
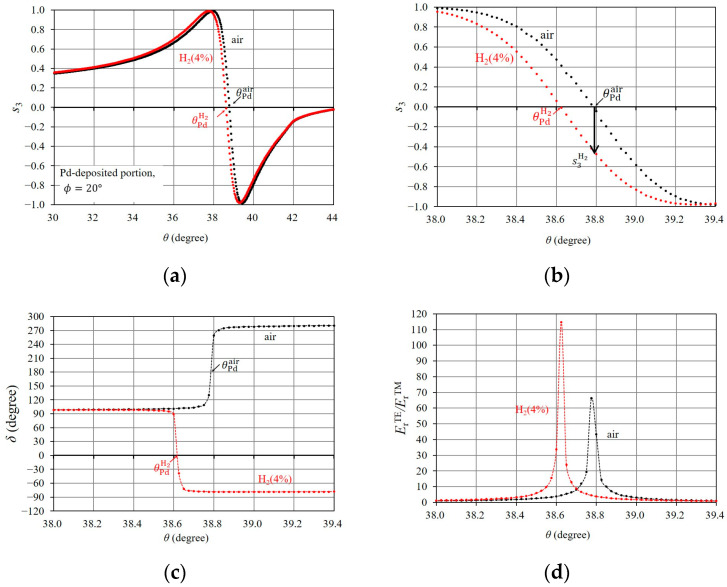
Effect of $H_{2}\left(4\%\right)$ exposure on rapid changes in $s_{3}$ in the Pd-deposited portion. (**a**) $s_{3}$ curves for $H_{2}\left(4\%\right)$ and air, (**b**) enlargement of area of rapid change in $s_{3}$, and (**c**) δ and (**d**) $E_{r}^{TE}/E_{r}^{TM}$ curves for $H_{2}\left(4\%\right)$ and air.

We examine the polarization property of the reflected light associated with SPR when the Pd-deposited portion, or the bare Al portion, is exposed to $H_{2}\left(4\%\right)$. Figure 8 shows the $s_{3}$ curves for air and $H_{2}\left(4\%\right)$ in the bare Al portion. The $s_{3}$ curve for $H_{2}\left(4\%\right)$ is almost identical to that for air, with a very slight difference around their resonance angles. Therefore, it was difficult to detect hydrogen gas with a concentration near the LEL (for instance, $H_{2}\left(4\%\right)$) using the rapid change in $s_{3}$ in the bare Al portion. Next, we describe the effect of the exposure of the Pd thin-film layer to $H_{2}\left(4\%\right)$ on the polarization property associated with SPR. Figure 9a shows the shift in the $s_{3}$ curve near the resonance angle due to $H_{2}\left(4\%\right)$ exposure, and Figure 9b clearly illustrates the difference between the rapid change in $s_{3}$ for $H_{2}\left(4\%\right)$ and air. The difference between the rapid change in $s_{3}$, which was caused by the change in the complex refractive index of the Pd thin-film layer upon exposure to $H_{2}\left(4\%\right)$, is explained with the resonance properties of δ and $E_{r}^{TE}/E_{r}^{TM},$ which are associated with SPR. As shown in Figure 9c,d, δ for $H_{2}\left(4\%\right)$ fluctuates from 90° to −90° via 0°, whereas δ for air fluctuates from 90° to 270° via 180°, and the peak value of $E_{r}^{TE}/E_{r}^{TM}$ for $H_{2}\left(4\%\right)$ is larger than that for air. Thus, the exposure of the Pd thin-film layer to $H_{2}\left(4\%\right)$ has a significant effect on the 180° phase shift of δ and the increase in $E_{r}^{TE}/E_{r}^{TM}$, resulting in the significant shift in the rapid change in $s_{3}$.

Here, we describe an efficient technique to detect hydrogen gas using the rapid change in $s_{3}$, as observed in the Pd-deposited portion. With SPR sensing, the resonance angle is typically measured to detect a change in the refractive index of a sample. However, as estimated in Figure 9b, the variation in $\theta_{Pd}$ is small for a change in hydrogen gas concentration near the LEL. This required a precise angle measurement to detect a variation in $\theta_{Pd}$. An alternative technique for measuring such a small variation in $\theta_{Pd}$ has been proposed [23], which utilizes the approximate linearity of the rapid change in $s_{3}$ around $\theta_{Pd}^{air}$. If a gas sample changes from air to $H_{2}\left(4\%\right)$ under the angle of incidence fixed at $\theta_{Pd}^{air}$, $s_{3}$ then fluctuates from 0 to $s_{3}^{H_{2}}$, as illustrated by the arrow in Figure 9b. Therefore, we can detect hydrogen gas with concentrations within the range of 0 to 4% in nitrogen by measuring $s_{3}$ at $\theta =\theta_{Pd}^{air}$.

#### 3.2.2. Variation in $s_{3}$ Due to Hydrogen Gas Exposure

Using the SPR sensing technique measuring $s_{3},$ as stated above, we carried out experiments to detect gas mixtures containing 1 to 4% hydrogen in nitrogen. We first determined the resonance angle $\theta_{Pd}^{air}$ from the $s_{3}$ curve for air, and then fixed the angle of incidence θ at $\theta_{Pd}^{air}$. The state where the chamber is filled with air, and θ is fixed at $\theta_{Pd}^{air},$ is referred to as the initial state.

**Figure 10:**
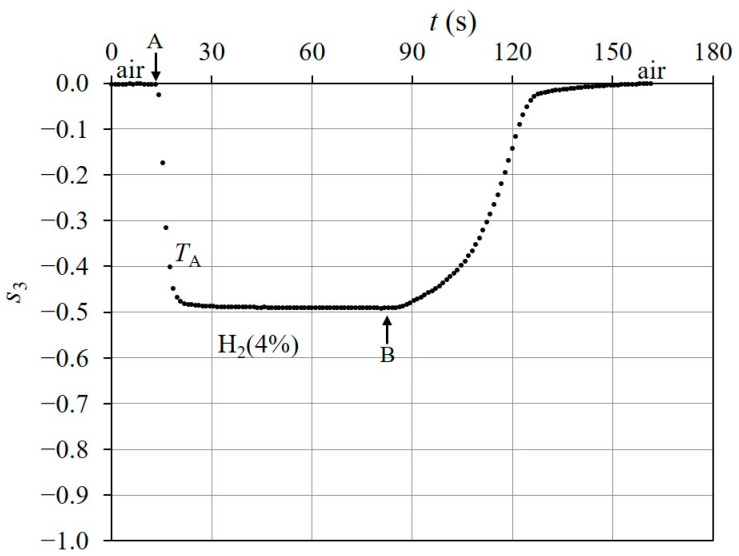
Time response of $s_{3}$ in the Pd-deposition portion for $H_{2}\left(4\%\right)$ exposure when θ is fixed at $\theta_{Pd}^{air}$. Injection of $H_{2}\left(4\%\right)$ into chamber started at t=13 s (point A) in its initial state and it stopped at t=83 s (point B). After the shutdown of B, $H_{2}\left(4\%\right)$ in the chamber dissipated into atmosphere.

**Figure 11:**
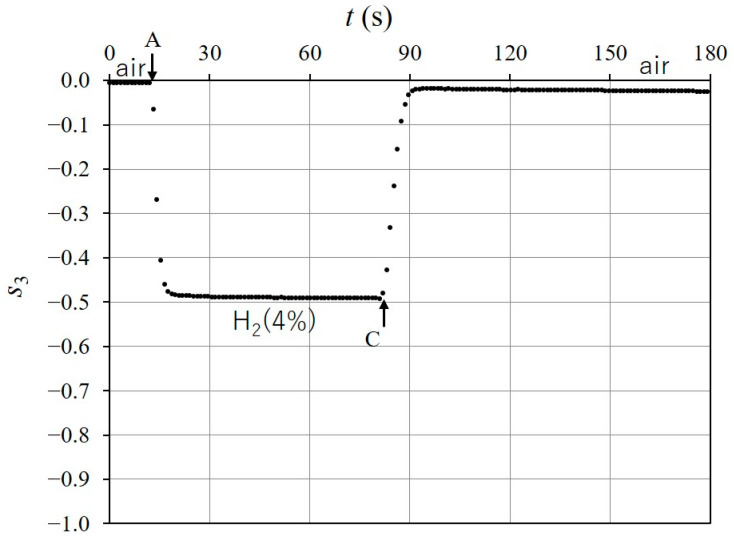
Recovery of $s_{3}$ to its initial state when the injection of $H_{2}\left(4\%\right)$ stopped at t=80 s (point C); immediately, the $H_{2}\left(4\%\right)$ in chamber was exhausted with the pump. It took 12 s from C to the stabilization of $s_{3}$.

**Figure 12:**
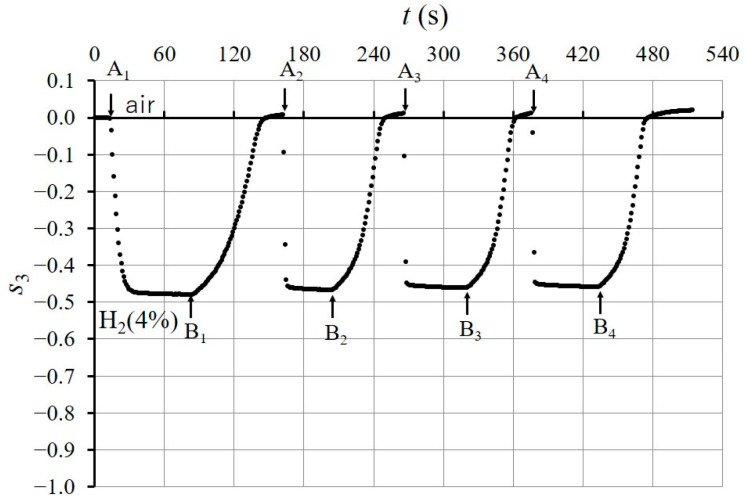
Repeatability of the time response of $s_{3}$ after four times of exposure to $H_{2}\left(4\%\right)$. Injection of $H_{2}\left(4\%\right)$ into the chamber started at A_1_ to A_4_, and stopped at B_1_ to B_4_. After each shutdown, the $H_{2}\left(4\%\right)$ in the chamber dissipated into atmosphere.

Figure 10 shows the time response of $s_{3}$ when the injection of $H_{2}\left(4\%\right)$ into the chamber started at t=13 s (point A on the figure) in the initial state and stopped at t = 83 s (point B). In Figure 10, the response value reaches −0.5, which is close to $s_{3}^{H_{2}}$ (see Figure 9b), and the response time is $T_{A}=5.5$ s. We used the response time, $T_{A},$ which was defined as the time necessary for the response to vary from the initial state to 90% of the total change [14]. After the shutdown of B, the $H_{2}\left(4\%\right)$ remaining in the chamber dissipated into the atmosphere, and $s_{3}$ returned to its initial state (air) in 75 s. If the $H_{2}\left(4\%\right)$ in the chamber is removed more quickly, $s_{3}$ returns to the initial state more quickly. Figure 11 shows that $s_{3}$ returned to its initial state in 12 s from the shutdown (point C on the figure), when $H_{2}\left(4\%\right)$ was exhausted with a pump. Figure 12 shows the time response of $s_{3}$ when the injection and dissipation of $H_{2}\left(4\%\right)$ was repeated four times in succession. $H_{2}\left(4\%\right)$ was injected into the chamber at A_1_ to A_4_ (shown in the figure) and $H_{2}\left(4\%\right)$ in the chamber dissipated into the atmosphere after the shutdown of B_1_ to B_4_. The time response of $s_{3}$ indicates good repeatability for the successive exposure of the Pd-deposited portion to $H_{2}\left(4\%\right)$.

**Figure 13:**
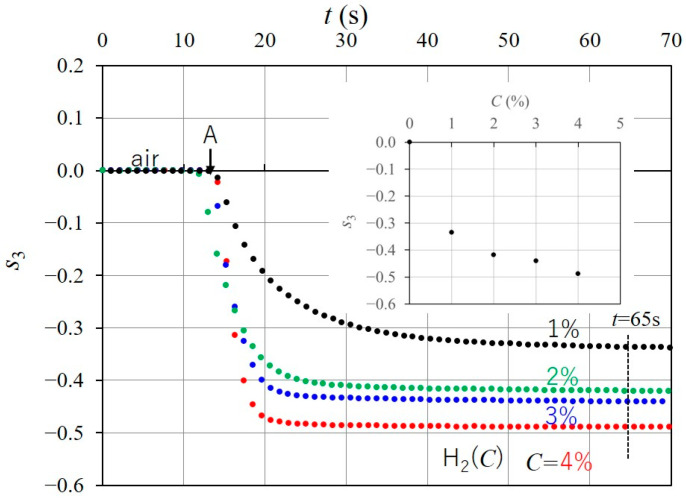
Time responses of $s_{3}$ for the four hydrogen gases, at different concentrations of 1, 2, 3, and 4%, in nitrogen. Inset plots response values $s_{3},$ at t=65 s, as a function of C.

We examined the response of $s_{3}$ to four hydrogen gases, with different concentrations in nitrogen, $H_{2}\left(C\right)\;C=1,\;2,\;3,and\;4\%$. Figure 13 shows the time responses of $s_{3}$ when each $H_{2}\left(C\right)$ was injected into the chamber at point A in the initial state, and the inset plots the value of $s_{3}$ at t=65 s, as a time response, as a function of C. The response value fluctuates significantly, reaching up to nearly 2%, but it fluctuates slowly above that level. This behavior, caused by the response to hydrogen gas concentration, which has been reported in the literature [9,11,14,35,36], is explained by the crystallographic phases of the palladium–hydrogen system in references [11,35]. Moreover, the time taken for $s_{3}$ to stabilize increases as C decreases [11].

#### 3.2.3. Discussion

As stated above, the SPR sensing technique, using the rapid change in $s_{3},$ in the Pd-deposited portion, provides a sensitive and stable response to the exposure of the Pd thin-film layer to gas mixtures containing hydrogen, at concentrations from 1 to 4%, in nitrogen. This demonstrates that the SPR sensing technique enables the detection of a small change in the complex refractive index of the Pd thin-film layer, which is caused by exposure to hydrogen gas. Therefore, the SPR sensing technique can be used to investigate the optical properties of a Pd thin-film layer exposed to hydrogen gas, with a concentration near the LEL.

However, there are some issues regarding the application of the SPR sensing technique, using the rapid change in $s_{3},$ to hydrogen sensors. The reduction in the recovery time, observed in Figure 11, is necessary for its application to hydrogen SPR sensors, in addition to improving the response time delay with a reduction in hydrogen gas concentration. Alloying Pd with gold [19], or creating multilayered structures such as Au/SiO_2_/Pd [12], may be effective in improving reaction and recovery times. Moreover, we observed a reduction in the response value of $s_{3},$ and a delay in response time regarding the experiments that were conducted after the Pd-deposited portion was repeatedly exposed to hydrogen gas. This suggests the degradation of the hydrogen detection performance of the Pd thin-film layer due to the mechanical damage of Pd upon exposure to hydrogen. Pd is susceptible to cracking, blistering, and delamination upon repeated exposure to hydrogen [17,37], and countermeasures to prevent mechanical damage (e.g., alloying of Pd with nickel [18] and gold [19] and capping of Pd with a gold layer [36]) have been reported. The SPR sensing technique, using the rapid change in $s_{3},$ may be available for hydrogen sensitive materials other than a Pd thin-film, such as Pd alloy/composite films [18,19,21] and multilayer films, including a Pd thin-film [12,36], which improve the performance of hydrogen gas detection.

## 4. Conclusions

We have investigated an efficient technique for detecting a small change in the complex refractive index of the Pd thin-film layer coated on the surface of an aluminum grating in a conical mounting. As a result, we revealed a rapid change in $s_{3},$ with a steep slope around the resonance angle, $\theta_{sp},$ at which point, SPR occurs in the Pd thin-film layer. The rapid change in $s_{3}$ results from both a variation in the phase and amplitude of the reflected light, which are strongly affected by SPR (i.e., a rapid phase shift in δ and a sharp increase in $E_{r}^{TE}/E_{r}^{TM})$. Therefore, the SPR sensing technique, using a rapid change in $s_{3},$ successfully detects hydrogen gas with a concentration near the LEL; $s_{3}$ in the vicinity of $\theta_{Pd}$ fluctuates significantly in response to a small change in the complex refractive index of the Pd thin-film layer upon exposure to the hydrogen gas.

As the polarization property associated with SPR occurs in the Pd thin-film coated aluminum grating, we investigated the rapid change in $s_{3}$ when the angle of incidence is varied at the fixed wavelength. Similarly, we predict that $s_{3}$ will change rapidly at the resonance wavelength at which SPR occurs, when the wavelength is varied at the fixed angle of incidence. The rapid change in $s_{3}$ around the resonance wavelength is a topic for future research.

## Figures and Tables

**Figure A2:**
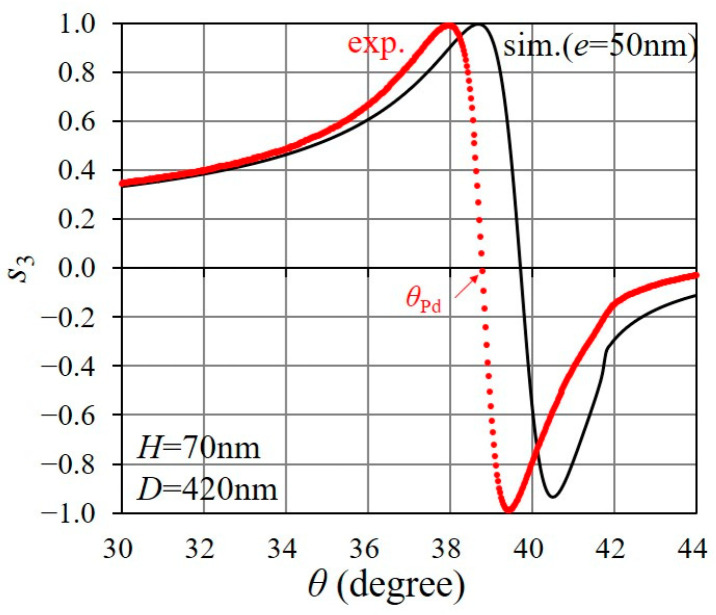
Comparison of rapid changes $s_{3}$ between the experiment and computer simulations.

## Data Availability

Data underlying the results presented in this paper are not publicly available at this time but may be obtained from the authors upon reasonable request.

## Appendix A

SPR in a metal grating, in a conical mounting, is numerically analyzed by solving the problem of plane–wave diffraction in a metal grating [31]. Using the numerical algorithm described in [31], we can simulate the polarization states of the reflected light from the Pd thin-film coated aluminum grating, as formulated in Section 2.2. The parameters required for the computer simulation concern information about the incidental light, the complex refractive indices of aluminum and Pd comprising the Pd-coated aluminum grating, and the profile of the Pd thin-film. For simplicity, we approximated the profiles of the upper and lower boundaries of the Pd thin-film using the same sinusoid with a corrugation depth of H=70 nm, and a period of d=417 nm, which is calculated using the groove density, at 2400 lines/mm. The incidental light used was a TM-polarized plane–wave with a wavelength of λ=672 nm. We used $n_{Al}=1.7116-j7.9108$ [38,39] and $n_{Pd}=1.9007-j4.3864$ [38,40], respectively, as the complex refractive indices of aluminum and Pd at λ=672 nm.

Figure A1 shows the reflectance r and normalized Stokes parameter $s_{3}$ of the reflected light from the Pd thin-film coated aluminum grating, with different Pd thin-film thicknesses e when the angle of incidence θ fluctuated under the azimuthal angle, which was fixed at ϕ=20°. The r and $s_{3}$ curves for e=0 nm corresponded with the bare aluminum grating; this highlights that the occurrence of SPR is associated with the excitation of surface plasmons along the surface of the aluminum grating in the conical mounting, due to the absorption dip in r and the rapid change in $s_{3}$ [23]. As e increases, SPR shifts to a lower incident angle side, with an increase in the peak-to-peak value of the $s_{3}$ curve, but above 45 nm (approximately), the SPR property becomes almost the same as that of the bare Pd grating. The increase in the peak-to-peak value suggests that the coupling between the incidental light and surface plasmons becomes stronger. The rapid change in $s_{3}$ for e=45 nm has a steep slope associated with sharp SPR, which can be available in hydrogen gas detection. Therefore, we numerically estimated 45 nm (approximately) as the Pd thin-film thickness to be coated on the aluminum grating.

To confirm the validity of the computer simulation, we compared the numerical result for the Pd thin-film coated aluminum grating with the experimental result in Figure A2. The experimental result is the $s_{3}$ curve for the Pd-deposited portion, shown in Figure 5b, and the numerical result was calculated from the same parameters as in Figure A1, except for e=50 nm and D=420 nm. We used D as 420 nm to match the cutoff of the −1st-order diffracted mode between the experimental and the numerical results. The numerical result shows the characteristics of the rapid change in $s_{3}$ well, which were obtained by the experiment, except for the slight deviation in the resonance angle.
